# Prevalence of back pain in employees of a German chemical company: results of a large cross-sectional study

**DOI:** 10.1186/s12995-019-0236-y

**Published:** 2019-05-28

**Authors:** Matthias Claus, Michael Schuster, Stefan Webendörfer, David A. Groneberg, Jacqueline Jähner, Daniel Schiffmann

**Affiliations:** 1 Corporate Health Management, FEH/CS – H308, BASF SE, 67056 Ludwigshafen am Rhein, Germany; 2 Institute of Occupational Medicine, Social Medicine and Environmental Medicine, Goethe University Frankfurt, 60590 Frankfurt am Main, Germany

**Keywords:** Back pain, Chemical company, Germany, Occupational medicine, Prevention, Disabling back pain, Prevalence, Multinomial logistic regression, Cross-sectional study

## Abstract

**Background:**

With the current study, we aimed to determine the prevalence of back pain in employees of a German chemical company. We put a specific focus on disabling back pain and its association with sociodemographic, lifestyle- and work-related characteristics.

**Methods:**

We used cross-sectional data, surveyed in health check-ups between 2011 and 2014 in Ludwigshafen am Rhein (Germany). A blood sample, physical examination and anamnesis by an occupational health physician as well as a written questionnaire were part of the check-up. A modified version of the *Standardised Nordic Musculoskeletal Questionnaire* was utilized to survey the back-pain specific information. Disabling back pain was defined as presence of any back pain in the past 12 months which prevented employees from carrying out their usual working tasks. We used multinomial logistic regression analysis to assess the association between (categorical) duration of disabling back pain and sociodemographic, lifestyle-, and work-related characteristics.

**Results:**

Overall, 17,351 employees participated in the health check-up, information on 16,792 persons could be used for analyses. Participants were on average 43.7 (SD: 9.7) years old and mainly male (79.1%). Lifetime-, 12-months-, and 7-days-prevalence of any type and duration of back pain were 72.5, 66.1, and 27.1% respectively. About one third (33.5%) had suffered from an episode of disabling back pain, 16.7% 1–7 days, 10.3% 8–30 days, and 6.5% more than 30 days. Multinomial regression analysis yielded that 8–30 days or more than 30 days of disabling back pain (relative to 0 days) were more likely with older age, female gender, being married, former or current smoking, lower occupational status, higher work-related stress score, history of a spinal injury, and diagnosed dorsopathy.

**Conclusions:**

There is a great need for action regarding multifaceted preventive measures and early interventions, especially for manual workers, older employees and women, where occupational medicine can play a decisive role.

## Background

Back pain remains a serious and wide-spread symptom of major public health concern in many industrialized countries [1, 2]. With regards to Germany, the lifetime-, one-year- and point prevalence of any type and duration of back pain, amount to 85, 76, and 34% respectively within the adult population [3]. Consequently, almost everyone experiences episodes of back pain throughout their lives. Besides direct adverse effects of back pain on people affected and their relatives, companies have to cope with reduced productivity of employees, workplace absenteeism, and early retirements due to incapacity for work [4, 5]. According to recent data published by the major German healthcare providers, back pain (ICD-10: M54) resulted in approximately 36 million days of workplace absence (7.3% of all days) [6] and 4293 early retirements due to incapacity for work (2.5% of all early retirements) [7] in the year 2015. The total average direct (medical) and indirect (economical; e.g. workplace absence) costs of back pain were estimated to amount to €1322 per person a year, corresponding to €49 billion annually for the whole adult German population [8].

In view of these profound individual and societal consequences, the prevention of back pain is of significant importance. In order to implement targeted preventive measures, it is necessary to identify those groups most severely affected by back pain.

With the current study, we aimed to determine the prevalence of back pain in employees of a German chemical company using data surveyed during voluntary occupational health check-ups. Due to its potentially highest impact on individuals and society, we put a specific focus on activity-limiting disabling back pain (DBP) and its association with sociodemographic, lifestyle- and work-related characteristics.

## Methods

### Study design

The present cross-sectional study is based on data surveyed during regular health check-ups at a chemical company in Ludwigshafen am Rhein (Germany) between January 1st, 2011 and December 31st, 2014. In addition to regular, mandatory occupational examinations, the company offers to its employees the possibility to participate in voluntary health check-ups regarding early detection of chronic diseases. This check-up was introduced in the year 2011 at the Ludwigshafen site and can be repeated every three years. Employees are invited to participate by a personal invitation letter via email. The health check-up consists of a venous blood sample, a comprehensive physical examination including anamnesis and documentation of health behavior by an occupational health physician, as well as completing a written questionnaire. The questionnaire includes, amongst others, an extensive module on back pain and work-related stress. The retrospective analysis of routine data gathered during the check-ups was approved by the ethical committee of the Medical Association of the German State of Rhineland-Palatinate.

### Back pain-related variables

Information regarding back pain was surveyed using a health check-up questionnaire. We used modified questions from the Standardised Nordic Questionnaire for the analysis of musculoskeletal symptoms [9]. Modifications were made regarding the type of back pain experienced. Whereas the Nordic Questionnaire focuses on “low back pain”, we did not specify a specific location of back pain but referred to back pain in general instead. The main variable of interest “*duration of disabling back pain*” was defined as the duration that back pain prevented from undertaking normal work activities (at home or at the workplace) during the last 12 months (0 days / 1–7 days / 8–30 days / more than 30 days). Further items included lifetime prevalence of back pain (“ever experienced back pain”), 12-months-prevalence (“duration of back pain during the last 12 months” [categorical]), 7-days prevalence (“experienced back pain in the last 7 days”), ever been hospitalized due to back pain, ever changed job or duties due to back pain, reduced ability to undertake work or leisure activities during the last 12 months due to back pain, ever visited a medical professional (doctor, physiotherapist, chiropractor or other such person) due to back pain, and ever acquired a spinal injury due to an accident. Furthermore, we considered history of spondylopathies (ICD 10-Code: M45-M49) and other types of dorsopathies (ICD 10-Code: M50-M53, excluding back pain) in our analyses, as documented by the responsible occupational health physician during examination/medical anamnesis.

### Sociodemographic, lifestyle- and work-related characteristics

Age (at time of examination), gender, occupational status (manual workers, skilled/supervisory workers, managerial staff), marital status (married, single, divorced, widowed), and working time system (day work, shift work [any type]) were directly extracted from employee data. Classification of occupational status into manual workers, skilled/supervisory workers and managerial staff represents roughly the socioeconomic status of the participants. The manual workers, working in the production lines, represent physical workers, skilled/supervisory workers usually have a more advanced education, often performing commercial activities or office work, while managerial staff hold an academic degree. Regarding shift workers, 90.2% worked 4x12h-rotating shifts, 6.8% 3x12h-rotating shifts, and 3.0% other types of shift (e.g. night shifts) at time of participation. A more detailed description of the types of shift work is available in the publication by Yong et al. (2015) [10].

Smoking status (smoker, former smoker, non-smoker), height and weight were ascertained during medical anamnesis by the responsible medical officer. Body-Mass-Index (BMI) was calculated using the formula *weight [in kg]/height [in m]*^*2*^ and was categorized according to the classification provided by the World Health Organization (normal weight: BMI < 25 / overweight: 25 ≤ BMI < 30 / obesity: BMI ≥ 30). Finally, we included a work-related stress-score in our analyses, based on five items which had to be answered on a five-point Likert-scale during the check-up. These items included the frequency of pressure of work-related deadlines being too high, the frequency of being overwhelmed by challenges at work, the frequency of thinking of work-related problems during leisure time, the frequency of negative impact of work-related stress on private life (“never” to “very often” for all of the previous items), and description of the workplace atmosphere (“very poor” to “very good”). All items were extracted from validated published instruments and have been used in the employees’ opinion survey in the company for several years [11]. An unweighted sum score of work-related stress (ranging from 0 [lowest stress] to 20 [highest stress]) was created from all items.

### Statistical analyses

We used absolute and relative frequencies (in case of categorical variables) and arithmetic mean with corresponding standard deviation (SD; in case of continuous variables) for a general description of sociodemographic, lifestyle- and work-related characteristics of the participants. Descriptive statistics and prevalence of DBP by sociodemographic, lifestyle- and work-related characteristics are presented for all participants and separately for manual workers, skilled/supervisory workers and managerial staff.

In order to assess the potential association of the ordinal-scaled dependent variable duration of DBP with sociodemographic, lifestyle- and work-related characteristics, ordered logistic regression analysis (proportional odds model) was considered. However, since the proportional odds assumption was violated, we decided to use multinomial logistic regression models. We used an iterative process of model building. First, all sociodemographic, lifestyle- and work-related characteristics, which were significantly (*p* < 0.05) associated with DBP in univariable analyses, were eligible to be included into the final multivariable model. Next, we fitted a preliminary multivariable model and inspected the importance of every included variable using Wald statistics. In case variables did not contribute significantly to this model (*p* ≥ 0.05), corresponding variables (BMI and working time system) were eliminated. In the following, the assumption of linearity in logits was examined for the continuous independent variables age and stress score using visual inspection of Lowess smoothed plots and Box-Tidwell-Tests. As this assumption appeared not to be met, categorical age (< 35 / 35–39 / 40–44 / 45–49 / 50–54 / ≥55) and quartiles of stress score (Q1 [lowest]: 0–6 points / Q2: 7–8 points / Q3: 9–10 points / Q4 [highest]: ≥11 points) were used in the multivariable model. Finally, we used Wald tests to test for overall significant interactions between the variables occupational status, age, sex, and stress score (quartiles). The results are presented as adjusted (multinomial) odds ratios (aOR) with corresponding 95% confidence intervals (95%-CI). We excluded observations from the regression analyses in case missing values were observed in one or more independent variables (< 3% of observations; complete case approach). *P*-values < 0.05 were considered statistically significant. As this was an exploratory analysis we did not adjust for multiple testing. All statistical analyses were carried out using STATA/SE Version 15.0 (StataCorp LLC, College Station, TX, USA).

## Results

Overall, 17,351 employees participated in the occupational health check-up. Exclusion of trainees (*n* = 70) and respondents with a missing/implausible answer regarding the dependent variable “duration of disabling back pain” (*n* = 489) led to a final sample of 16,792 respondents. Study participants were on average 43.7 (SD: 9.7) years old and mainly male (79.1%). One third (33.2%) were employed as manual workers, 45.8% as skilled/supervisory workers, and 21.0% as managerial staff. Detailed information regarding sociodemographic, lifestyle- and work-related characteristics of all respondents and stratified by occupational status can be found in Table 1.

**Table 1:** Absolute and relative frequencies of sociodemographic, lifestyle- and work-related characteristics in all participants of the occupational health check-up (2011–2014) and stratified by occupational status (*n* = 16,792)

	Total	Manual workers	Skilled/supervisory workers	Managerial staff
	n (%)	n (%)	n (%)	n (%)
Total	16,792 (100.0)	5571 (100.0)	7690 (100.0)	3531 (100.0)
*Sociodemographic characteristics*				
Age (mean [SD])	43.7 (9.7)	43.1 (10.1)	43.4 (9.7)	45.2 (8.6)
< 35	3273 (19.5)	1165 (20.9)	1618 (21.0)	490 (13.9)
35–39	1858 (11.1)	634 (11.4)	730 (9.5)	494 (14.0)
40–44	2801 (16.7)	930 (16.7)	1250 (16.3)	621 (17.6)
45–49	3427 (20.4)	1078 (19.4)	1732 (22.5)	617 (17.5)
50–54	3312 (19.7)	1100 (19.8)	1463 (19.0)	749 (21.2)
≥ 55	2121 (12.6)	664 (11.9)	897 (11.7)	560 (15.9)
Gender				
Male	13,279 (79.1)	5317 (95.4)	5193 (67.5)	2769 (78.4)
Female	3513 (20.9)	254 (4.6)	2497 (32.5)	762 (21.6)
Marital status				
Single	3412 (20.3)	1343 (24.1)	1518 (19.7)	551 (15.6)
Married	11,980 (71.3)	3775 (67.8)	5422 (70.5)	2783 (78.8)
Divorced	1235 (7.4)	407 (7.3)	667 (8.7)	161 (4.6)
Widowed	129 (0.8)	33 (0.6)	75 (1.0)	21 (0.6)
Missing information	36 (0.2)	13 (0.2)	8 (0.1)	15 (0.4)
*Lifestyle-related characteristics*				
Body-Mass-Index (mean [SD])	26.6 (4.4)	27.9 (4.5)	26.5 (4.5)	25.0 (3.6)
Normal weight (< 25 kg/m^2^)	6442 (38.4)	1440 (25.9)	3073 (40.0)	1929 (54.6)
Overweight (25- < 30 kg/m^2^)	7153 (42.6)	2672 (48.0)	3191 (41.5)	1290 (36.5)
Obesity (≥30 kg/m^2^)	3186 (19.0)	1459 (26.2)	1423 (18.5)	304 (8.6)
Missing information	11 (0.1)	0 (0.0)	3 (0.0)	8 (0.2)
Smoking status				
Non-smoker	9087 (54.1)	2140 (38.4)	4176 (54.3)	2771 (78.5)
Former smoker	4206 (25.1)	1639 (29.4)	2044 (26.6)	523 (14.8)
Smoker	3454 (20.6)	1791 (32.2)	1453 (18.9)	210 (6.0)
Missing information	45 (0.3)	1 (0.0)	17 (0.2)	27 (0.8)
*Work-related characteristics*				
Working time system				
Day work	12,105 (72.1)	2248 (40.4)	6341 (82.5)	3516 (99.6)
Shift work	4637 (27.6)	3323 (59.7)	1309 (17.0)	5 (0.1)
Missing information / other	50 (0.3)	0 (0.0)	40 (0.5)	10 (0.3)
Work-related stress score in quartiles (mean [SD])	8.2 (2.9)	7.7 (2.9)	8.3 (2.9)	8.9 (2.8)
Q1 (0–6 points)	4704 (28.0)	1878 (33.7)	2144 (27.9)	682 (19.3)
Q2 (7–8 points)	4448 (26.5)	1525 (27.4)	2005 (26.1)	918 (26.0)
Q3 (9–10 points)	3792 (22.6)	1144 (20.5)	1772 (23.0)	876 (24.8)
Q4 (≥11 points)	3539 (21.1)	903 (16.2)	1632 (21.2)	1004 (28.4)
Missing information	309 (1.8)	121 (2.2)	137 (1.8)	51 (1.4)

All percentages depicted in the table are column percentages; SD: standard deviation

About one third (33.5%) of respondents suffered from an episode of DBP in the year prior to participation, 16.7% 1–7 days, 10.3% 8–30 days, 6.5% more than 30 days (Table 2).

**Table 2:** Back pain in all participants of the occupational health check-up (2011–2014) and stratified by occupational status (*n* = 16,792)

	Total (*n* = 16,792)	Manual workers (*n* = 5571)	Skilled/supervisory workers (*n* = 7690)	Managerial staff (*n* = 3531)
	%	%	%	%
Ever experienced back pain				
No	27.5	28.0	25.6	30.9
Yes	72.5	72.0	74.4	69.1
Ever been hospitalized due to back pain				
No	92.1	90.1	92.8	93.9
Yes	7.7	9.7	7.0	6.1
Ever acquired a spinal injury due to an accident				
No	95.9	96.1	95.3	96.9
Yes	3.7	3.5	4.3	2.8
Ever changed job or duties due to back pain				
No	98.6	97.6	98.8	99.6
Yes	1.3	2.2	1.0	0.4
Duration of back pain during the last 12 months				
0 days	33.9	33.6	31.1	40.8
1–7 days	25.0	23.6	24.9	27.5
8–30 days	21.7	21.9	22.6	19.3
More than 30 days, but not every day	15.8	17.4	17.5	9.8
Every day	3.6	3.7	4.0	2.7
Reduced ability to undertake work activities (at home or at the workplace) during the last 12 months due to back pain				
No	84.8	79.6	84.8	93.1
Yes	14.5	19.5	14.7	6.2
Reduced ability to undertake leisure activities during the last 12 months due to back pain				
No	73.1	70.3	72.0	79.8
Yes	25.4	27.0	26.9	19.3
Duration that back pain prevented from undertaking normal work activities (at home or at the workplace) during the last 12 months				
0 days	66.6	60.0	65.4	79.7
1–7 days	16.7	17.8	17.4	13.3
8–30 days	10.3	13.4	10.7	4.5
More than 30 days	6.5	8.9	6.5	2.6
Ever visited a medical professional (doctor, physiotherapist, chiropractor or other such person) due to back pain				
No	55.2	54.3	53.4	60.8
Yes	44.2	45.3	46.1	38.5
Experienced back pain in the last 7 days				
No	72.2	73.3	69.0	77.4
Yes	27.1	26.3	30.1	21.8
History of spondylopathy (ICD-10: M45-M49)				
No	98.5	98.2	98.3	99.3
Yes	1.5	1.8	1.7	0.7
History of other dorsopathy (ICD-10: M50-M53) excluding back pain				
No	81.4	74.5	81.7	91.5
Yes	18.6	25.5	18.3	8.6

All percentages depicted in the table are column percentages. Relative frequencies of missing values have been omitted in the table

Lifetime prevalence (“ever experienced back pain”), 12-months prevalence (“duration of back pain during the last 12 months”), and 7-days prevalence (“experienced back pain in the last 7 days”) of any type and duration of back pain were 72.5, 66.1, and 27.1% respectively. Due to back pain, 44.2% have visited a medical professional, 7.7% have been hospitalized, and 1.3% had to change their jobs or duties. For all items, prevalence was lowest in managerial staff.

**Fig. 1:**
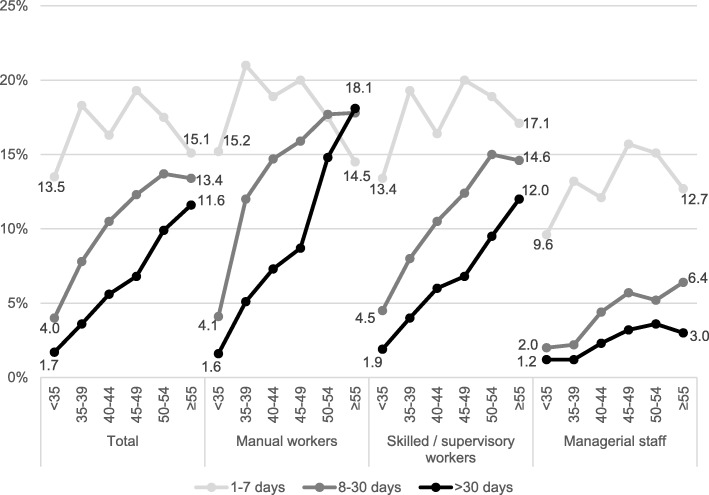
Duration of disabling back pain in the last 12 months by age group in all participants of the occupational health check-up (2011–2014) and stratified by occupational status (*n* = 16,792)

Regarding age, the 12-months-prevalence of any duration of DBP increases from 19.2 to 40.1% when moving from the lowest (< 35) to the highest (≥55) age category (Fig. 1).

With respect to differences between occupational groups, the proportion of respondents with more than 30 days of DBP increases from 1.6 to 18.1% in manual workers, 1.9 to 12.0% in skilled/supervisory workers, and 1.2 to 3.0% in managerial staff when moving from the youngest (< 35) to the oldest (≥55) age group.

Regarding characteristics other than age (Table 3), the 12-months-prevalence of DBP was comparatively high in divorced (42.2%) and obese (38.9%) respondents, former smokers (39.8%), employees with a high stress score (45.0%), respondents with a history of a spinal injury (55.5%), and those with a history of spondylopathy (69.3%) or other type of dorsopathy (58.5%).

**Table 3:** Duration of disabling back pain in the last 12 months in all participants of the occupational health check-up (2011–2014) and stratified by occupational status (*n* = 16,792)

	Total (*n* = 16,792)			Manual workers (*n* = 5571)			Skilled/super-visory workers (*n* = 7690)			Managerial staff (*n* = 3531)		
	1–7 days	8–30 days	> 30 days	1–7 days	8–30 days	> 30 days	1–7 days	8–30 days	> 30 days	1–7 days	8–30 days	> 30 days
	%	%	%	%	%	%	%	%	%	%	%	%
Total	16.7	10.3	6.5	17.8	13.4	8.9	17.4	10.7	6.5	13.3	4.5	2.6
*Sociodemographic characteristics*												
Gender												
Male	17.0	10.8	6.6	17.8	13.7	8.9	18.2	11.1	6.4	13.1	4.8	2.4
Female	15.4	8.3	6.0	16.5	7.1	9.1	15.7	10.0	6.6	13.9	3.4	3.0
Marital status												
Single	14.5	6.4	3.6	15.6	8.3	4.8	14.5	6.3	3.0	12.0	1.8	2.5
Married	17.0	11.0	7.1	18.2	14.8	10.3	18.0	11.7	7.3	13.3	4.7	2.3
Divorced	20.2	13.9	8.1	20.9	17.9	9.6	20.5	12.7	7.7	17.4	8.1	6.2
Widowed	11.6	16.3	12.4	15.2	12.1	15.2	10.7	17.3	10.7	9.5	19.1	14.3
*Lifestyle-related characteristics*												
Body-Mass-Index												
Normal weight (< 25 kg/m^2^)	15.1	7.5	4.8	16.7	10.2	7.1	16.0	8.5	5.3	12.6	3.8	2.4
Overweight (25- < 30 kg/m^2^)	17.3	11.7	7.4	17.7	15.0	9.6	18.5	11.5	7.4	13.7	5.0	2.6
Obesity (≥ 30 kg/m^2^)	18.3	12.9	7.7	18.9	13.4	9.4	18.2	13.8	7.0	15.8	5.6	3.3
Smoking status												
Non-smoker	16.0	8.3	4.4	18.9	11.5	6.6	16.9	9.8	4.7	12.3	3.7	2.3
Former smoker	17.5	13.1	9.2	16.1	16.3	11.2	18.5	11.9	9.0	18.0	7.7	3.8
Smoker	17.4	12.2	8.6	17.9	13.0	9.6	17.3	12.0	8.1	13.8	6.2	3.3
*Work-related characteristics*												
Working time system												
Day work	16.3	9.3	5.8	17.9	13.9	9.3	17.5	10.3	6.3	^a^	^a^	^a^
Shift work	17.6	13.0	8.3	17.7	13.0	8.7	17.4	12.9	7.3	^a^	^a^	^a^
Work-related stress score												
Q1 (0–6 points)	13.2	6.6	3.8	15.2	8.5	4.4	13.1	6.5	4.1	8.2	2.1	1.2
Q2 (7–8 points)	16.6	9.3	5.2	17.8	12.5	7.6	17.9	9.6	4.9	11.6	3.5	1.7
Q3 (9–10 points)	18.0	12.0	7.6	18.5	16.5	12.2	19.6	12.4	7.1	13.8	5.4	2.5
Q4 (≥ 11 points)	20.1	14.5	10.4	23.0	20.9	16.3	20.2	15.9	10.9	17.4	6.5	4.3
*Back-related variables*												
Ever acquired a spinal injury due to an accident												
No	16.5	10.0	6.0	17.5	13.0	8.5	17.3	10.4	5.9	13.0	4.3	2.5
Yes	21.6	17.5	16.4	23.8	22.3	20.2	20.4	17.1	17.4	21.4	9.2	5.1
History of spondylopathy (ICD-10: M45-M49)												
No	16.6	10.1	6.1	17.8	13.1	8.5	17.4	10.6	6.1	13.2	4.5	2.5
Yes	18.3	23.0	28.0	17.4	28.6	33.7	17.9	21.6	27.6	24.0	8.0	8.0
History of other dorsopathy excluding back pain (ICD-10: M50-M53)												
No	15.7	8.1	3.9	16.9	10.0	5.2	16.4	9.0	4.2	12.7	3.9	1.6
Yes	20.9	19.9	17.7	20.2	23.3	19.9	21.9	18.5	16.6	19.5	10.3	12.6

^a^Due to the low number of managerial staff working shifts (*n* = 5), prevalence of disabling back pain regarding working time system has been omitted for this group; prevalence for missing categories has been omitted in the table. All percentages depicted in the table are row percentages

In univariable multinomial logistic regression analyses (results not shown), all considered variables were significantly associated with DBP and thus eligible to be entered into the final multivariable model. Working time system and BMI however, did not contribute significantly to the multivariable model and were eliminated during the iterative process of model building. Results of the multivariable multinomial logistic regression analysis regarding the association between sociodemographic, lifestyle- and work-related characteristics with duration of DBP in the past 12 months are displayed in Table 4.

**Table 4:** Results of multivariable multinomial logistic regression analysis regarding the association of duration of disabling back pain with sociodemographic, lifestyle- and work-related factors (*n* = 16,346)

	Multivariable analysis (*n* = 16,346)		
	1–7 days vs. 0 days	8–30 days vs. 0 days	> 30 days vs. 0 days
*Sociodemographic characteristics*	aOR (95%-CI)	aOR (95%-CI)	aOR (95%-CI)
Age			
< 35 (reference)			
35–39	1.38 (1.17–1.63)	1.88 (1.45–2.44)	1.78 (1.21–2.61)
40–44	1.23 (1.05–1.43)	2.41 (1.91–3.03)	2.81 (2.02–3.90)
45–49	1.50 (1.29–1.73)	2.81 (2.25–3.51)	3.41 (2.47–4.70)
50–54	1.42 (1.22–1.66)	3.22 (2.57–4.04)	4.88 (3.55–6.70)
≥ 55	1.23 (1.03–1.47)	3.07 (2.40–3.92)	5.48 (3.92–7.65)
Gender			
Male (reference)			
Female	1.02 (0.90–1.14)	1.19 (1.02–1.39)	1.86 (1.54–2.24)
Marital status			
Single (reference)			
Married	1.17 (1.04–1.32)	1.32 (1.11–1.56)	1.26 (1.01–1.56)
Divorced	1.41 (1.17–1.70)	1.49 (1.18–1.89)	1.20 (0.88–1.63)
Widowed	0.83 (0.47–1.48)	1.70 (0.99–2.90)	1.47 (0.79–2.75)
*Lifestyle-related characteristics*			
Smoking status			
Non-smoker (reference)			
Former smoker	1.08 (0.97–1.21)	1.25 (1.10–1.43)	1.55 (1.32–1.82)
Smoker	1.07 (0.95–1.20)	1.20 (1.04–1.38)	1.51 (1.27–1.81)
*Work-related characteristics*			
Occupational status			
Managerial staff (reference)			
Skilled/supervisory worker	1.65 (1.46–1.86)	2.90 (2.41–3.50)	2.72 (2.13–3.47)
Manual worker	1.93 (1.68–2.20)	4.29 (3.53–5.23)	4.70 (3.64–6.07)
Work-related stress score (quartiles)			
Q1 (0–6 points; reference)			
Q2 (7–8 points)	1.41 (1.25–1.59)	1.62 (1.38–1.91)	1.64 (1.33–2.03)
Q3 (9–10 points)	1.70 (1.50–1.93)	2.28 (1.94–2.67)	2.61 (2.12–3.21)
Q4 (≥ 11 points)	2.22 (1.96–2.52)	3.34 (2.85–3.92)	4.33 (3.53–5.30)
*Back-related variables*			
Ever acquired a spinal injury due to an accident			
No (reference)			
Yes	1.89 (1.52–2.36)	2.63 (2.06–3.35)	4.18 (3.22–5.44)
History of spondylopathy (ICD-10: M45-M49)			
No (reference)			
Yes	2.00 (1.37–2.91)	3.24 (2.24–4.69)	5.59 (3.86–8.10)
History of other dorsopathy excluding back pain (ICD-10: M50-M53)			
No (reference)			
Yes	1.98 (1.77–2.21)	2.98 (2.63–3.36)	5.12 (4.43–5.90)

*aOR* adjusted (multinomial) Odds Ratio, *95%-CI* 95%-Confidence interval; Working time system and Body-Mass-Index were eligible to be entered into the final multivariable regression model but eliminated during the iterative process of model building

The odds of suffering from 8 to 30 days or > 30 days of DBP in the past year (relative to 0 days) are comparatively higher for older respondents, women, married participants, former and current smokers, skilled/supervisory and manual workers, employees with higher levels of stress, and those with a history of a spinal injury, spondylopathy or other type of dorsopathy. No significant interactions between occupational status, age, gender, and stress score were found.

## Discussion

### Main findings

With this cross-sectional study, we aimed to determine the prevalence of back pain in employees of a chemical company with a specific focus on activity-limiting DBP and its association with sociodemographic, lifestyle- and work-related characteristics. Lifetime-, 12-months-, and 7-days-prevalence of any type and duration of back pain were 72.5, 66.1, and 27.1% respectively. About one third (33.5%) suffered from an episode of DBP in the past 12 months, 16.7% 1–7 days, 10.3% 8–30 days, and 6.5% more than 30 days. DBP was associated with older age, female gender, being married, former or current smoking, lower occupational status, higher work-related stress score, history of a spinal injury, and diagnosed spondylopathy or other dorsopathy.

### Limitations

There are several limitations in our manuscript which should be acknowledged. First, results are limited by the fact that we used a cross-sectional study design. Thus, no conclusions regarding potential causal relationships between DBP and independent variables can be drawn. Furthermore, the possibility of selection bias has to be acknowledged. During the considered period of subject recruitment (2011–2014), 37,866 people (trainees and higher management excluded) were at some time employed at the Ludwigshafen site of the company. Thus, more than 50% of all eligible employees did not participate in the occupational health check-up. On the one hand, employees leading a healthy lifestyle might have been more willing to participate in the occupational health check due to a higher interest in health-related subjects. On the other hand, respondents with a poor health, who would not normally visit their general practitioner on a routine basis, may have accepted the opportunity to receive a health check at work. Depending on the type of selection, prevalence of back pain could be under- or overestimated in our sample. Furthermore, information on back pain relies entirely on the self-report by employees and is not based on an objectively verified examination by a medical expert. There is a possibility for recall bias if respondents were not able to correctly remember episodes of (disabling) back pain in the past year. A further limitation concerns the fact that information on duration and type of back pain treatment (e.g. surgical vs. conservative) of the participants was unknown, potentially underestimating the true prevalence of back pain and confounding the association of sociodemographic, lifestyle- and work-related factors with DBP. Additionally, there are further sociodemographic, lifestyle- and/or work-related factors potentially associated with back pain (e.g. physical activity) which have not been considered in our analyses.

### Implications

As mentioned by other authors as well, studies on back pain differ widely according to the location of pain (e.g. low back vs. back in general), composition of the surveyed population, and type of prevalence considered [12, 13]. Thus, comparing our findings with earlier national and international studies must be done with caution. With regard to Germany, results from the German Back Pain Survey on a subsample of employed respondents (*n* = 4412; mean age: 42y), carried out between 2002 and 2006, the 12-months prevalence of any type and duration of back pain was higher in both genders compared to our investigation (79.3% vs. 68.9% in women; 76.7% vs. 65.3% in men) [14]. In the same study, lifetime prevalence of back pain was provided for all participants only (including unemployed and retirees; *n* = 9263) and amounted to 85.7% in women and 85.3% in men [3] (vs. 73.4 and 72.2% respectively in our study). Results from the German National Health Survey on a subgroup of employed participants (*n* = 3488; 18-69y) carried out between 1997 and 1999 by the Robert Koch Institute yielded a 12-months- and 7-days-prevalence of any type and duration of back pain of 60.0% (62% females / 58% males) and 34.4% (38% females / 32% males) respectively [15] which is comparable to the findings in our study of 66.1% (69% females / 65% males) and 27.1% (34% females / 25% males). In a further survey on the German adult general population from the year 2003 (*n* = 8318; 18-79y), the 12-months-prevalence of any type of back pain exceeded 60% in women and 50% in men in all age groups [13]. Another German back pain study conducted in 2003 (*n* = 1456; 25-74y), found that the (age and education-adjusted) 12-months-prevalence was 74.3% in females and 73.4% in males [16]. Regarding DBP, Schmidt and colleagues reported a three-months-prevalence of 9.2% for the German adult general population based on the Graded Chronic Pain Scale, which combines disability points and pain intensity [3, 17]. Differences in the definition and prevalence period however impede a direct comparison of this figure with the 12-months-prevalence of DBP in our study (33.5%).

Regarding international studies, two recent systematic reviews aimed to provide global estimates on the prevalence of low back pain [1, 18]. The review by Hoy et al. (2012) included 165 studies from 54 countries and found (unadjusted) mean point, 12-months, and lifetime prevalence proportions of (any type of) low back pain of 18.3, 38.0, and 38.9% respectively [1]. Based on a comparatively small subset of studies, the point prevalence of activity-limiting low back pain in the same review was 17.0% [1]. According to the authors, these figures have to be interpreted with caution since significant methodological heterogeneity was present between studies [1]. The review by Meucci et al. (2015) found a prevalence of chronic low back pain (lasting at least six weeks) of 19.6% for individuals between 20 and 59 years old based on 15 cross-sectional studies with a response rate greater than 75% [18]. A further (narrative) review article by Manchikanti et al. (2014) additionally summarized the evidence on low back pain in an occupational context and found that 28% of the US industrial population suffer from disabling back pain at some time, and that 9–26% of all US industry insurance claims are related to occupational low back pain according to state-by-state surveys [19].

Comparing our findings with the German general population, there is no evidence that prevalence of back pain in our employees is higher. However, the fact that one in three participants suffered from episodes of activity-limiting DBP during the last 12 months is certainly challenging and points to an enormous potential for prevention. In this regard, the profound differences in back pain prevalence according to sociodemographic, lifestyle- and work-related characteristics could help prioritizing interventions on those groups most severely affected. Older employees certainly form one of those groups, with more than 40% of respondents ≥ 55 years suffering episodes of DBP in the last 12 months. This finding is all the more critical since demographic shifts and associated aging workforces point to a further increase in the prevalence of back pain in the near future. Other studies have pointed to this association as well [3, 12, 20], although, a curvilinear relationship between age and back pain with a certain decline after peaking between 50 and 59 [13] or 40–69 years [1] has also been described. In a systematic review on age and back pain prevalence however, curvilinear relationships were found for benign and mixed forms of back pain only, while severe back pain continued to increase with increasing age [21].

Regarding gender, the prevalence of any duration of DBP in the past 12 months was higher in men than women (34.4% vs. 29.7%). However, the odds of 8–30 days or > 30 days of DBP (relative to 0 days) were significantly higher in women in the multivariable regression analysis. Most national and international studies identified women to be more frequently affected by any type of back pain [1, 3, 12–14, 18, 20, 22]. A variety of potentially interacting biological (e.g. differences in sex hormones, genotype, muscle/bone mass), psychosocial (e.g. differences in disclosure of symptoms due to sociocultural beliefs on masculinity/femininity) and exposure-related mechanisms (e.g. double workday [domestic tasks and regular work] in women / heavier physical work in men) underlying gender disparities in pain have been discussed in recent reviews [1, 18, 23]. Concerning marital status, married employees had significantly higher odds of DBP (relative to singles) across all cut-off values of the dependent variable. Although similar associations were found in other studies [3, 24] as well, this finding cannot be explained easily. One explanation could be that married respondents have (a higher number of) children and are thereby exposed to mechanical activities associated with child rearing (e.g. carrying and lifting of children) [24].

In addition to the aforementioned characteristics, there was a strong gradient regarding occupational status. The relative frequency of any duration of DBP was lowest in managerial staff (20.4%) and continuously increased from 34.6% in skilled/supervisory workers to 40.1% in manual workers. Consistent results were found in multivariable analysis, with odds of more than 30 days of back pain for manual workers and skilled/supervisory workers being 4.70 and 2.72-times the odds of managerial staff respectively. The association of back pain with low socioeconomic status, measured either by a single item or by combining occupational status, income and education to a score, has also been shown in a variety of studies [3, 12–15, 18, 25–27]. The most plausible explanation for the finding in our study might be that physically demanding occupational tasks associated with back pain, such as repeated manual carrying, holding, and lifting of heavy weights, working in uncomfortable postures or occupational exposure to whole-body vibration, are more prevalent in lower occupational groups [15, 19, 28]. Other explanations include problematic health behaviors (e.g. lower participation in preventive offers such as back-specific trainings), reduced knowledge about possibilities regarding improvement of back health, and decreased access to medical care in lower socioeconomic classes [12, 26]. Additionally, in workers with sub acute or chronic low back pain, strong evidence for a negative association of low sociodemographic status with return to work has been found in a recent systematic review by Steenstra and colleagues [29].

We further identified work-related stress as being associated with DBP. The prevalence of DBP increased continuously from 23.6 to 45.0% when moving from the lowest to the highest quartile of the stress score. In multivariable analysis, odds of 8–30 or more than 30 days DBP in the highest quartile of the stress score were 3.34- and 4.33-times respectively the odds of DBP in the lowest. Evidence regarding the association of psychosocial factors at work and back pain has been summarized in two systematic reviews by Hoogendoorn et al. (2000) [30], and Hartvigsen et al. (2004) [31], with conflicting results. Whereas Hoogendoorn and colleagues conclude that there is evidence for an association between psychosocial occupational factors and back pain [30], Hartvigsen et al. conclude that there is moderate evidence for no such association [31]. Additionally, it is important to note that back pain can be both, a cause and effect of (occupational) stress, or as Schneider et al. suggest, the association is “fundamentally bidirectional” [15].

Concerning lifestyle-related factors, current and former smoking were also associated with DBP. There was a similar finding in the study by Neuhauser et al. (2005), where daily smokers had significantly higher odds of back pain in the last 12-months after multivariable adjustment [13]. In addition to that, six studies included in the review by Meucci et al. assessed the association of smoking and prevalence of chronic low back pain. In all studies, smokers, as compared to non-smokers, had a higher prevalence of low back pain. The authors suggest an accelerated joint degeneration process and an increased potential of pain impulse transmission in the central nervous system caused by nicotine as a potential explanation for this finding [18]. However, in a systematic review on smoking and low back pain based on 47 studies, the association between smoking and back pain was summarized as inconsistent across studies and, when present, generally weak and visible only in large studies [32]. The authors concluded that smoking is rather a weak risk indicator for back pain than a causal factor for its development [32].

In order to prevent back pain, several measures like physical exercise, education or workplace interventions can be considered. Current evidence on preventive measures was summarized in a recent systematic review and meta-analysis by Steffens and colleagues (2016) including 23 published reports on more than 30,000 participants. The authors conclude that exercise alone or combined with education is effective for preventing low back pain while education alone or other measures including back belts, shoe insoles or ergonomic adjustments are probably not [33]. Maher et al. add in their recent seminar paper that exercise to be effective requires a substantial commitment by the participants in terms of time, and stress the importance of extending the scope of physical exercise beyond the back on the inclusion of the upper and lower limbs and overall fitness [34].

## Conclusions

The prevalence of back pain can be considered as high in our study, with one out of three employees affected from activity-limiting disabling pain in the past 12 months. As a consequence of our investigation, there is a great need for action regarding multifaceted preventive measures and early interventions, especially for manual workers, older employees and women, where occupational medicine might play a decisive role. Future investigations, focusing on the success of different preventive measures in the occupational context are necessary in order to monitor a potential improvement in the burden caused by back pain.

## Data Availability

The datasets generated and/or analyzed during the current study are not publicly available due to company-specific data-protection laws.
